# The bactericidal effect of vancomycin is not altered by tranexamic acid, adrenalin, dexamethasone, or lidocaine in vitro

**DOI:** 10.1038/s41598-021-90302-7

**Published:** 2021-05-24

**Authors:** Christiane Schwerdt, Eric Röhner, Sabrina Böhle, Benjamin Jacob, Georg Matziolis

**Affiliations:** Orthopaedic Professorship of the University Hospital Jena, Campus Eisenberg, Klosterlausnitzer Str. 81, 07607 Eisenberg, Germany

**Keywords:** Antimicrobials, Clinical microbiology

## Abstract

One of the most challenging complications of total knee arthroplasty (TKA) is periprosthetic joint infection (PJI). There is growing evidence of a good anti-infective effect of intrawound vancomycin powder in total joint arthroplasty. At the same time, various different locally applied substances have become popular in total joint arthroplasty. The objective of this study was therefore to investigate a possible inhibition of the bactericidal effect of vancomycin by tranexamic acid, adrenalin, lidocaine, or dexamethasone. The bactericidal effect of vancomycin was quantified using the established method of the agar diffusion test. The plates were incubated with *Staphylococcus aureus* or *Staphylococcus epidermidis* and four wells were stamped out. The wells were filled with vancomycin alone, the tested substance alone or a mixture of the two. The fourth well remained empty as a control. The plates were incubated overnight at 37 °C and the zone of inhibition in each field was measured on the next day. All tests were run three times for each pathogen and mean values and standard deviations of the measurements were calculated. Differences between the substances were tested using the t-test at a level of significance of 0.05. The bacterial growth was homogeneous on all plates. The baseline value for the zone of inhibition of vancomycin was on average 6.2 ± 0.4 mm for *Staphylococcus aureus* and 12 ± 0.3 mm for *Staphylococcus epidermidis*. In all other substances, no inhibition was detected around the well. The combination of vancomycin and each other substance did not show any different result compared to vancomycin alone. The bactericidal effect of vancomycin on *staphylococci* is not altered by tranexamic acid, adrenalin, dexamethasone, or lidocaine in vitro.

## Introduction

One of the most challenging complications of total knee arthroplasty (TKA) is periprosthetic joint infection (PJI). Preoperative antibiotic prophylaxis has become a standard procedure to reduce the infection rate^1^. Additionally, especially in spine surgery, intrawound application of antibiotics (e.g. vancomycin) has been shown to be effective in preventing infections since more than a decade ago^2, 3^. There is now growing evidence of a comparable anti-infective effect of intrawound vancomycin powder in total joint arthroplasty^4–6^. In contrast to spine surgery, different locally applied substances have become popular in total joint arthroplasty since the introduction of fast track surgery and enhanced recovery protocols. Vasoconstrictors (e.g. adrenalin) and tranexamic acid are given to reduce postoperative bleeding, local anesthetics (e.g. lidocaine) are used for pain management, and glucocorticoids (e.g. dexamethasone) are administered to reduce postoperative inflammation. There is broad evidence of positive effects for all of these substances alone and in combination^7–11^. On the other hand there is no data about interactions of locally applied vancomycin with any of these substances. Although there is clinical evidence that combination therapy of locally applied vancomycin and tranexamic acid does not affect the rate of infections, no experimental study primarily focusing on the interaction of vancomycin with any of the above mentioned substances is known to us^12, 13^.Vancomycin may be deactivated by a changed pH value, a chemical reaction or another antagonization by tranexamic acid, adrenalin, lidocaine, or dexamethasone.

The objective of this study was therefore to investigate a possible inhibition of the bactericidal effect of vancomycin by these substances in vitro.

## Materials and methods

The bactericidal effect of vancomycin was quantified using the established method of the agar diffusion test^14^. A solid nutritional agar medium was produced on a plate and four fields were marked. The most common pathogens in PJI (*Staphylococcus aureus* and *Staphylococcus epidermidis*) were used for testing^15–17^. The plates were incubated with *Staphylococcus aureus* (100 µl, DSM 346) or *Staphylococcus epidermidi*s (100 µl, DSM 1798) and a well was stamped out in each field. Three well were filled as given in Table 1.

**Table 1:** Allocation of the substances on the plates.

1st well	2nd well	3rd well
Vancomycin(50 mg/ml)	Vancomycin + tranexamic acid	Tranexamic acid(100 mg/ml)
Vancomycin(50 mg/ml)	Vancomycin + lidocaine	Lidocaine(1%)
Vancomycin(50 mg/ml)	Vancomycin + dexamethasone	Dexamethasone(4 mg/ml)
Vancomycin(50 mg/ml)	Vancomycin + adrenalin	Adrenalin(1 mg/ml)

The wells were filled with vancomycin (50 mg/ml), TXA (100 mg/ml) or TXA and vancomycin. The fourth well or field remained empty as a control. The same procedure was repeated with adrenalin (1 mg/ml), dexamethasone (4 mg/ml) and lidocaine (1%). The plates were placed in an incubator at 37 °C overnight. The zone of inhibition in each field was measured on the next day. Sufficient agar diffusion capacity for the experiments was confirmed by exemplary broth dilution assay for each of the tested substances.

All tests were run three times for each pathogen and mean values and standard deviations of the measurements were calculated. Differences between the substances were tested using the t-test at a level of significance of 0.05.

## Results

The bacterial growth was homogeneous on all plates. The baseline value for the zone of inhibition of vancomycin was on average around 6 mm for *Staphylococcus aureus* and 12 mm for *Staphylococcus epidermidis*. In all other substances, no inhibition was detected around the well (Table 2). The combination of vancomycin and each other substance did not show any different result compared to vancomycin alone.

**Table 2:** Zone of inhibition of the different substances in mm. TXA, adrenalin, dexamethasone, and lidocaine did not inhibit bacterial growth at all.

	Vancomycin	Vancomycin + TXA	Vancomycin + adrenalin	Vancomycin + dexamethasone	Vancomycin + lidocaine
*Staph. aureus*	6.2 ± 0.4	6.3 ± 0.6	6.3 ± 0.6	6.3 ± 0.6	6.0 ± 0.0
*Staph. epidermidis*	12 ± 0.3	11.8 ± 0.3	12.2 ± 0.3	12.0 ± 0.5	11.8 ± 0.3

## Discussion

The main result of this study is that the bactericidal effect of vancomycin is not compromised by TXA, adrenalin, dexamethasone, or lidocaine in vitro. The prophylactic effect of locally applied vancomycin should therefore not be influenced by these similarly locally administered substances.

Vancomycin is a glycopeptide antibiotic (GPA) produced by the actinomycete *Amycolatopsis orientalis*. The first introduction in hospitals was in 1958. Structurally, it shows aromatic amino acids with contents such as sugar residues, chlorine atoms and lipid chains, which have undergone extensive oxidative cross-linking to build a core heptapeptide scaffold. Additionally, there are proteinogenic and non-proteinogenic amino acids contained in the main antimicrobial GPA scaffold. Five of these residues are aromatic and two are aliphatic. The three oxidative cross-links between the aromatic amino acids in vancomycin lead to the structural conformation representing the binding pocket for the cellular antibiotic target^18^.

GPA are bactericidal by inhibiting bacterial cell wall synthesis in the late extracellular stages of PG cross-linking. Binding to the D-Ala-D-Ala dipeptide terminus of the peptidoglycan (PG) precursors, vancomycin sequestrates the substrate from transpeptidation and transglycosylation reactions^19, 20^.

In a systematic review and meta-analysis, Heckmann et al. compared six retrospective studies between the year 2017 and 2018 about intrawound usage of vancomycin in primary and revision TKA and THA. Although the analysis quality was low, they showed that intrawound vancomycin may reduce the risk of PJI. But they also point out the need for randomized controlled trials^21^. The first study was a retrospective consecutive case series by Otte et al., in which 1640 patients with primary or revision TKA or THA where included. The respective antibiotic group received 1 g vancomycin intracapsularly, the control group none. Both groups were monitored for a follow-up of three months using the musculoskeletal infection society definition of PJI^22^. The rate of PJI was 0.49% in the vancomycin group and 1.5% in the control group^5^. Winkler et al. had the same study design. The only differences where the smaller number of patients (744), the higher dose of intracapsular vancomycin (2 g), and the longer follow-up, at over six months. In general, they had a higher infection rate in both groups compared to Otte et al. In the vancomycin group the infection rate was 2.66% and in the control group 7.55%^6^. Patel et al. also performed a retrospective consecutive case series, but only with 460 patients and moreover only primary TKA or THA^4^. The antibiotic group received 1 g vancomycin intra- and extracapsularly. The follow-up was longer than three months, but they used the same definition as the others for PJI. Patel et al. had a higher infection rate in the vancomycin treated group at 0.57% and also in the control group at 2.68% compared to Otte. The last retrospective consecutive case series included in the meta-analysis of Heckmann was published by Dial et al. in 2018^23^. They included 265 patients who had undergone primary THA. Like Patel, they administered 1 g of vancomycin intra- and extracapsularly and followed them for over three months. However, they used a different definition of PJI (International Consensus Meeting on Periprosthetic Joint Infections)^24^. Dial et al. showed an infection rate of 0.7% in the vancomycin group and 5.5% in the control^23^. Both values are lower than in Patel´s case series. Also mentioned in the meta-analysis was the retrospective case–control published by Riesgo et al. in 2018^25^. They investigated a small number of patients (74) with revision TKA and THA for over one year. During surgery, the patients in the antibiotic group received an injection with 1 g vancomycin subfascially and 1 g subcutaneously. Riesgo et al. also used the Musculoskeletal Infection Society definition of PJI. There were much higher infection rates in both groups: vancomycin group 16.67% and control group 36.84%, maybe suggesting that there is in general an increasing infection rate in revision total arthroplasty. The last trial included was a retrospective cohort study on 115 patients with primary TKA performed by Khatri et al.^26^. They applied 1 g of vancomycin intracapsularly and had a follow-up of over six months. One negative aspect is that there was no specific definition of PJI. Furthermore, they had high infection rates for primary TKA: 9.8% in the antibiotic group and 12.5% in the control group.

In their systematic review/meta-analysis Xu et al. concluded that “intrawound vancomycin used in primary hip and knee arthroplasty may reduce incidence of PJI” and that “intrawound vancomycin may increase risk of aseptic wound complications”^27^.

In 2014, Alshryda et al. published a systematic review and meta-analysis about topical use of tranexamic acid in TJA of the hip and knee, including 11 randomized controlled trials (RCT) of TXA in TKA, two RCT of TXA in THA and one RCT of TXA in both^28^. They explored blood loss, rate of transfusion and thromboembolic events after topical application of TXA during THA and TKA. The rate of blood transfusions was significantly decreased by topical TXA risk ratio (RR): 4.51; 95% confidence interval (CI): 3.02 to 6.72; *p* < 0.001 in TKA and RR: 2.56; 95% CI: 1.32 to 4.97, p = 0.004 in THA. They found no differences between the rate of thromboembolic events in topical usage of TXA and a placebo. Moreover, there was a benefit of topical compared to intravenous administration of TXA in placebo controlled trials^28^. Also, in a meta-analysis, Yu et al. showed a significant reduction of perioperative blood loss, hemoglobin drop and transfusion rate by using a combination of locally applied adrenalin and TXA, compared with topical TXA alone^11^. Furthermore, there was no increase in incidence of deep venous thrombosis and hematoma with the combination of TXA and epinephrine.

Kerr et al. achieved significant results in pain reduction without systemic side-effects after TKA with local infiltration analgesia^9^. They used a combination of ropivacaine, adrenalin and ketorolac (non-steroidal, anti-inflammatory drug). Also in arthroscopic knee surgery there are significant results regarding better pain reduction with a combination of intraarticular injection of dexamethasone and local anesthetics (e.g. bupivacaine), as shown by an Indian group in 2014^29^. An older publication by Dahl et al. also showed a reduction of pain in knee arthroscopy through intraarticular application of lidocaine and epinephrine^30^. This reflects the many years’ clinical practice and proven efficacy of intraarticular injection of the substances tested. However cytotoxicity of vancomycin, tranexamic acid and local anesthetics could be demonstrated by several studies and combination therapy would be very likely to increase local cytotoxic effects^31–33^.

There are several limitations of this study. First, this was an *invitro* study, so that interactions of the tested substances with joint fluid, cells and the whole organism may change the results. We can assume that the combination with glucocorticoids would modulate the immune system in a way that the bactericidal effect of vancomycin could be compromised in vivo. Additionally, antimicrobial activity of lidocaine and attenuating inflammatory response of tranexamic acid were described^34, 35^. Second, no chemical analysis was performed, so that—highly improbably—chemical reactions cannot be ruled out. However, even under these conditions, a bactericidal effect was evident. Third, only the effect of vancomycin on *staphylococci* was tested, based on the fact that these are the most common pathogens in PJI. A different result on other organisms is again improbable but not impossible.

The bactericidal effect of vancomycin on *staphylococci* is not altered by tranexamic acid, adrenalin, dexamethasone, or lidocaine in vitro. The prophylactic effect of intraarticularly applied vancomycin should therefore not be influenced by these substances in vivo.
